# The association between antigenemia, histology with immunohistochemistry, and mucosal PCR in the diagnosis of ulcerative colitis with concomitant human cytomegalovirus infection

**DOI:** 10.1007/s00535-022-01931-2

**Published:** 2022-10-26

**Authors:** Tsukasa Yamawaka, Hiroki Kitamoto, Masanori Nojima, Tomoe Kazama, Kohei Wagatsuma, Keisuke Ishigami, Shuji Yamamoto, Yusuke Honzawa, Minoru Matsuura, Hiroshi Seno, Hiroshi Nakase

**Affiliations:** 1 Department of Gastroenterology and Hepatology, Sapporo Medical University School of Medicine, S-1, W-16, Chuo-Ku, Sapporo, 060-8543 Japan; 2 Department of Gastroenterology and Hepatology, Kyoto University School of Medicine, Kyoto, Japan; 3 Center for Translational Research, Institute of Medical Science Hospital, University of Tokyo, Tokyo, Japan; 4 Department of Gastroenterology and Hepatology, Kyorin University School of Medicine, Tokyo, Japan

**Keywords:** Ulcerative colitis, Human cytomegalovirus, Mucosal PCR

## Abstract

**Background:**

Human cytomegalovirus (HCMV) colitis can be involved in active ulcerative colitis (UC) in patients refractory to steroid and immunosuppressive drugs. Histological examination with colonic biopsy specimens and antigenemia assays are the standard tests for diagnosing HCMV enterocolitis, and we have previously reported the usefulness of mucosal polymerase chain reaction (PCR) methods. However, the associations among histopathological tests, antigenemia assays, and mucosal PCR are unknown.

**Methods:**

We retrospectively analyzed 82 UC patients who underwent mucosal biopsy from inflamed colonic tissues for histological evaluation and mucosal PCR to detect HCMV. We analyzed the relationships between the HCMV-DNA copy number in colonic mucosa and other HCMV tests.

**Results:**

In total, 131 HCMV mucosal PCR tests from 82 UC patients were positive. The HCMV-DNA copy number was significantly higher in patients with positive immunohistochemistry (IHC) (*p* < 0.01) and was correlated with the number of positive cells for the antigenemia (C7-HRP, *p* < 0.01; C10/11, *p* < 0.01). Receiver operating characteristic curve analysis confirmed 1300 copies/μg of HCMV-DNA as the best diagnostic cut-off value to predict positive results of antigenemia (area under the curve = 0.80, 95% CI 0.68–0.93). HCMV-DNA copy number also correlated with the total UCEIS score (*p* = 0.013) and the bleeding score (*p* = 0.014). For each individual patient, a positive correlation between the change in total UCEIS score and HCMV-DNA copy number was observed (*p* = 0.040).

**Conclusion:**

The antigenemia assay and histopathological test with IHC were significantly associated with the HCMV-DNA copy number in colonic tissues. Moreover, endoscopic examination with the UCEIS can help diagnose the HCMV colitis in UC patients.

**Supplementary Information:**

The online version contains supplementary material available at 10.1007/s00535-022-01931-2.

## Introduction

Human cytomegalovirus (HCMV), which is a member of the herpesvirus family, is generally contracted during childhood and can persist as a lifelong latent infection [1, 2]. Latent HCMV is reactivated under inflammatory conditions in immunosuppressed hosts and can cause organ damage [3]. Much attention has been given to HCMV infection in patients with ulcerative colitis (UC) who are refractory to steroids and immunosuppressive drugs as well as to those with *Clostridioides difficile* infection because HCMV infection causes significant morbidity in UC patients [4, 5]. Therefore, an early diagnosis of HCMV infection in patients with refractory UC and rapid initiation of antiviral agents could help to avoid colectomy.

Confirming the presence of HCMV on biopsy is the gold standard for the diagnosis of HCMV enterocolitis [1]. Diagnosis by histological examination has a high specificity (92–100%) but a variable sensitivity (10 to 87%) [6, 7]. The HCMV antigenemia assay is also widely used for the detection of HCMV antigen-positive neutrophils in peripheral blood using C7-HRP and C10/11 monoclonal antibodies against the primary structural antigen (pp65) of HCMV. This method has a sensitivity of 47.0–65.4% and a specificity of 81.7–93.6% for diagnosing HCMV gastrointestinal disease [8–11].

The usefulness of the mucosal polymerase chain reaction (PCR) method with colon biopsy tissues for diagnosing HCMV infection and determination of the treatment strategy for patients with refractory UC has been demonstrated in previous studies [12–16]. Consistent with the clinical research, several reports have indicated that the copy number of HCMV-DNA in colonic tissues contributes to the decision in favor of antiviral treatment [12, 14]. However, the mucosal PCR method is not available in all medical institutions, and data on the associations among histopathological findings, antigenemia assays, and mucosal HCMV-PCR are limited. In this study, we aimed to clarify the relationships between the HCMV-DNA copy number in colonic mucosa and other HCMV tests or endoscopic score.

## Methods

### Patients

We retrospectively analyzed a total of 82 patients with UC who were suspected of concomitant HCMV colitis and underwent HCMV mucosal PCR at Kyoto University Hospital (78) or Sapporo Medical University Hospital (4) between October 2013 and March 2020. The diagnosis of UC was based on clinical, endoscopic, radiologic, and histologic parameters. Fecal bacterial culture showed no specific pathogens in any of the enrolled patients. We used antiviral therapy when the diagnosis of HCMV infection was compatible based on the results of antigenemia and histopathological tests, endoscopic findings, viral titers by mucosal PCR, and clinical manifestations. Antiviral agents were administered to the patients intravenously or orally for 2–4 weeks and ended after observing clinical improvement. In this study, we posted information about this study on the hospital website, gave participants the opportunity to opt out, and considered that patients who did not opt out provided tacit consent for study participation.

### Study design

The primary outcome was the association between HCMV mucosal PCR and other HCMV tests. The secondary outcomes were the association between HCMV mucosal PCR and the patient characteristics, endoscopic findings, and rate of surgery after HCMV mucosal PCR. Clinical data were obtained retrospectively from electronic medical records.

### Assessment of endoscopic severity

All 82 patients underwent colonoscopy. The endoscopists categorized the disease phenotype based on the Montreal classification (E1/E2/E3) and assessed endoscopic severity according to the Mayo endoscopic subscore (MES) and Ulcerative Colitis Endoscopic Index of Severity (UCEIS). The items composed of the UCEIS (erosion and ulcers, vascular pattern, and bleeding) were also assessed (Table S1) [17]. Although the endoscopic score was not independently assessed by central reviewers, it was evaluated by IBD experts at each facility.

### Histopathology

We took colonic biopsy specimens from the margins of the ulcer or the most severely inflamed mucosa of the left colon or rectum in patients without ulcerations. They were fixed in formalin and embedded in paraffin and then evaluated with hematoxylin and eosin (H&E) staining and immunohistochemistry (IHC) using anti-HCMV monoclonal antibodies (Dako Cytomation, Kyoto, Japan).

### HCMV antigenemia assay

The HCMV antigenemia assay directly detects HCMV antigen-positive neutrophils present in peripheral blood using C7-HRP and C10/11 monoclonal antibody (SRL Inc., Tokyo, Japan) against the primary structural antigen of HCMV. A positive result in this assay was defined as one or more HCMV-positive cells per 50,000 leukocytes applied.

### Quantitative real-time PCR (mucosal PCR)

We extracted DNA from colonic tissue with the QIAamp DNA Mini Kit (QIAGEN, Tokyo) according to the manufacturer’s instructions, and detected HCMV-DNA using quantitative real time PCR. This assay was performed using an ABI Prism 7700 Sequence Detector System (Perkin Elmer Applied Biosystems, Foster City, CA, USA). The oligonucleotide primers used for HCMV-DNA amplification were 5_-GACTAGTGTGATGCTGGCCAAG-3_(forward) and 5_-GCTACAATAGCCTCTTCCTCATCTG-3_(reverse), and the 6-carboxyfluorescein-labeled probe was 5_-AGCCTGAGGTTATCAGTGTAATGAAGCGCC-3_. The PCR conditions were 95 °C for 10 min, 50 cycles at 95 °C for 15 s and 62 °C for 1 min as described previously [12, 13]. Ten or more copies/μg of HCMV-DNA was defined as positive in this study [12]. For cases performing multiple mucosal PCR tests, we separately counted the data of each test.

### Statistical analysis

Statistical analysis was performed using IBM SPSS Statistics 25 (IBM SPSS, Chicago, USA). The value of the HCMV-DNA copy number was logarithmically transformed for the statistical analysis (ln HCMV-DNA). Categorical and continuous data were compared using the Mann–Whitney *U* test and Spearman’s rank correlation coefficient, as appropriate, to calculate the statistical significance of the demographic and clinical variables according to the manufacturer’s instructions. A value of *p* < 0.05 was considered statistically significant.

## Results

### Baseline clinical features of the patients

A total of 82 patients with 131 HCMV mucosal PCR tests were enrolled. The median age was 48 years (range 17–90) and the sample consisted of 46 males and 36 females. The median MES and total UCEIS values were 2 and 5, respectively (Table 1). Regarding the extent of disease according to the Montreal classification, the number of patients with E1, E2, and E3 was 0, 31, and 100, respectively. Among all enrolled patients, 75.6% were treated with prednisolone (PSL), 48.9% with immunomodulator (IM), 19.8% with tacrolimus (Tac), and 17.6% with biologics (Bio). The positive rates of tests other than HCMV mucosal PCR were 3.5% (4/115) for nuclear inclusion with H&E staining, 22.2% (14/63) for IHC, and 20.9% (9/43) for HCMV antigenemia using C7-HRP and 41.3% (19/46) using C10/11; IHC, C7-HRP, and C10/11 had many missing values (Table 2).

**Table 1:** Baseline patient characteristics and clinical features

Man, *n* (%)	46 (56)^†^
Age, years, median (IQR) [range]	48 (31–66) [17–90]
Montreal classification^§^, *n* (%)	
E1 E2 E3	0 (0) 31 (24) 100 (76)
Endoscopic findings	
MES (0 / 1 / 2/ 3) Total UCEIS (2 / 3 / 4 / 5 / 6 / 7)	0 / 3 / 44 / 84 3 / 4 / 34 / 60 / 24 / 6
Medication^¶^, *n* (%)	
IM Tac Bio	64 (48.9) 26 (19.8) 23 (17.6)
PSL alone + IM + Tac + Bio All	27 (20.6) 61 (46.6) 25 (19.1) 20 (15.3) 99 (75.6)
Antiviral therapy (%)	51 (38.9)
Performed colectomy, *n* (%)	13 (9.9)0

*PCR* polymerase chain reaction, *MES* Mayo endoscopic subscore, *UCEIS* ulcerative colitis endoscopic index of severity, *PSL* prednisolone, *IM* immunomodulator; *Tac* tacrolimus, *Bio* biologics

^†^82 UC patients with a total of 131 positive ^†^PCR tests were enrolled

^‡^HCMV-DNA of 10 copies/μg or more

^§^Montreal classification: the subgroups of UC defined by extent are:

Ulcerative proctitis (E1): involvement limited to the rectum

Left-sided UC (E2): involvement limited to the portion of the colorectum distal to the splenic flexure

Extensive UC (E3): involvement extends proximal to the splenic flexure

^¶^Medications used at the time when the mucosal PCR was performed. Multiple selections are allowed

**Table 2:** The positive rates of histopathology (H&E/IHC), antigenemia assay for HCMV

	Positive rate
Histopathology	
H&E staining IHC	3.5% (4/115) 22.2% (14/63)
Antigenemia assay	
C7-HRP C10/11	20.9% (9/43) 41.3% (19/46)

*H&E* hematoxylin and eosin, *IHC* immunohistochemistry, *HCMV* human cytomegalovirus, *HRP* horseradish peroxidase

82 UC patients with a total of 131 ^†^positive PCR tests

^†^HCMV-DNA of 10 copies/μg or more

Missing data: HE staining (*n* = 16), IHC (*n* = 68), C7-HRP (*n* = 88), C10/11 (*n* = 85)

### Relationship between the HCMV-DNA copy number and clinical features

**Fig. 1:**
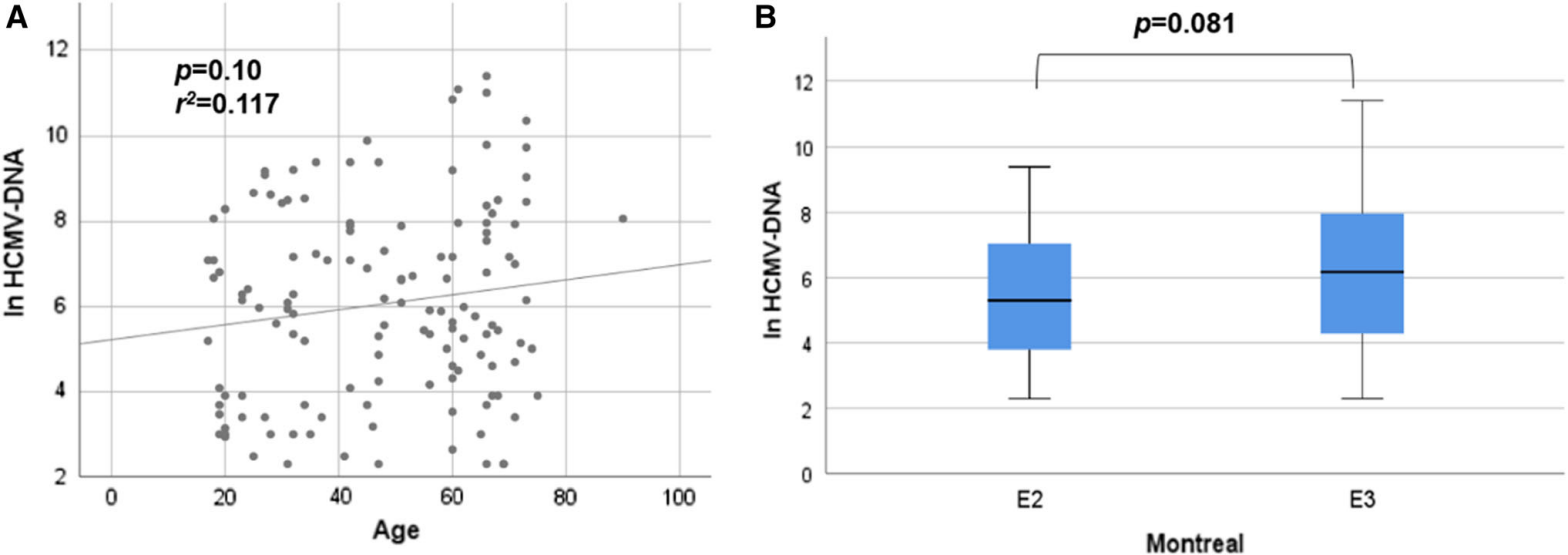
The relationship between (**A**) Age, (**B**) ^†^Montreal classification, and HCMV-DNA copy number. The vertical axis is the natural log-transformed copy number of HCMV-DNA (ln HCMV-DNA). **A** Pearson correlations were used to study the association between ln HCMV-DNA and Age. The *P* value and the correlation coefficient were *p* = 0.10 and *r*^*2*^ = 0.117, respectively. **B** Non-parametric Mann–Whitney test was used to compare ln HCMV-DNA between E2 and E3 (no case of E1). ^†^Montreal classification: The subgroups of UC defined by extent are: ulcerative proctitis (E1): involvement limited to the rectum. Left-sided UC (E2): involvement limited to the portion of the colorectum distal to the splenic flexure. Extensive UC (E3): involvement extends proximal to the splenic flexure. *HCMV* human cytomegalovirus

We investigated several factors associated with the HCMV-DNA copy number in colonic mucosa. The HCMV-DNA copy number tended to increase with age (Fig. 1A). Regarding the extent of disease, patients with Montreal E3 had a higher HCMV-DNA copy number than those with E2 (Fig. 1B). Regarding medication, patients treated with PSL had a higher HCMV-DNA copy number than those without PSL, although there was no significant difference (Fig. S1A, B). Other medical treatments, such as IM, Tac, and Bio, did not affect the HCMV-DNA copy number (Fig. S1C-E).

### Relationship between the HCMV-DNA copy number and other HCMV tests

**Fig. 2:**
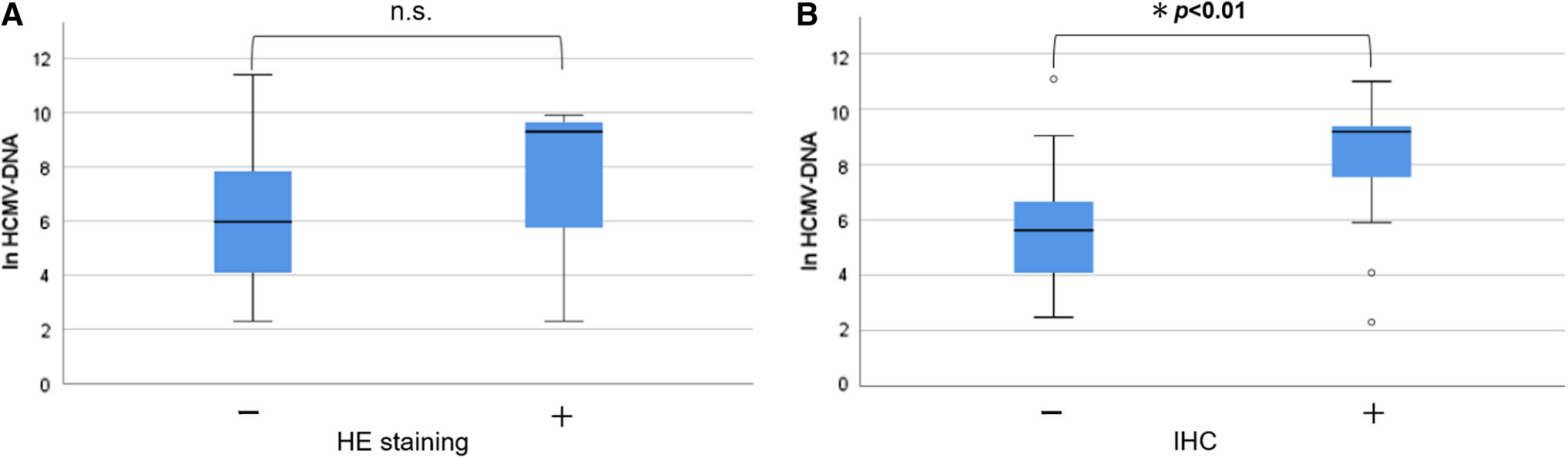
The relationship between histopathological examination and HCMV mucosal PCR copy number. **A** Hematoxylin and eosin (H&E) staining and **B** immunohistochemistry using anti-HCMV monoclonal antibody. The vertical axis is the natural log-transformed copy number of HCMV-DNA (ln HCMV-DNA). *Significant at *p* < 0.01 by non-parametric Mann–Whitney *U* test. *HCMV* human cytomegalovirus, *HE* Hematoxylin and Eosin staining, *IHC* Immunohistochemistry, *n.s.* not statistically significant

**Fig. 3:**
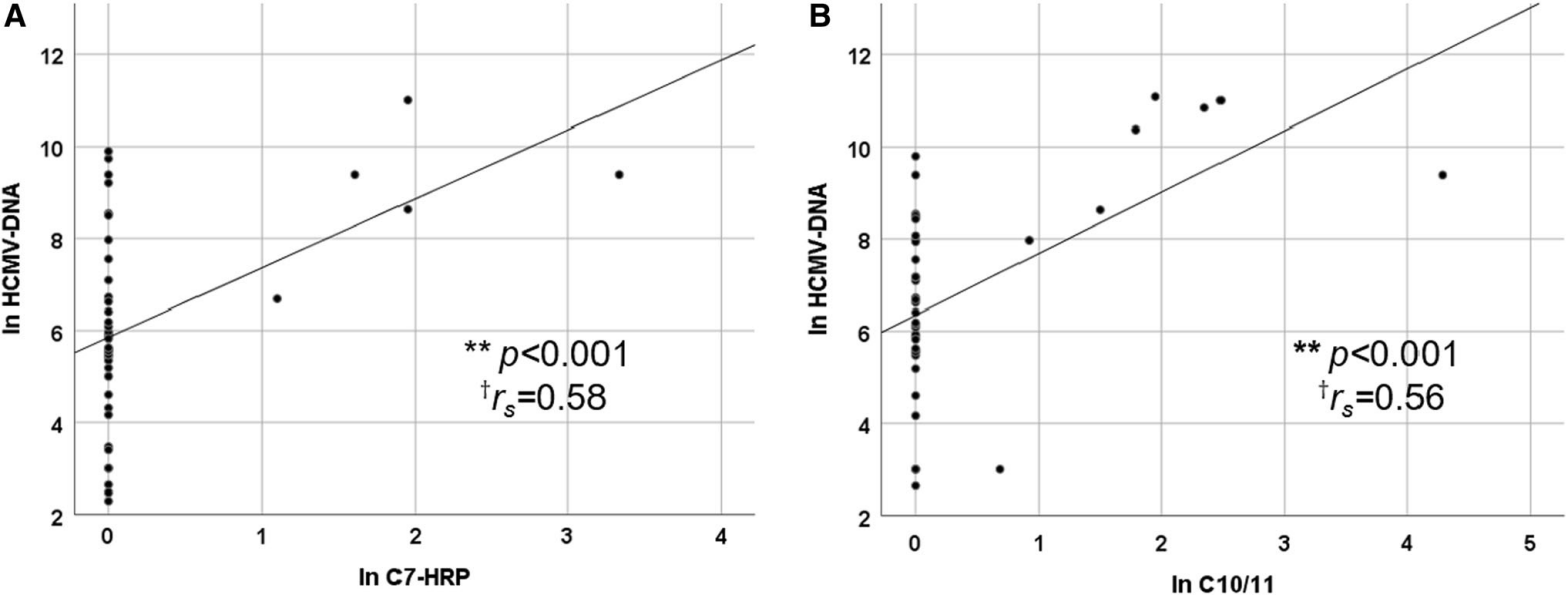
The relationship between **A** C7-HRP, **B** C10/11 antigenemia assay and HCMV-DNA copy number. The vertical axis is the natural log-transformed copy number of HCMV-DNA, and the horizontal axis is the natural log-transformed number of C7-HRP or C10/11 positive cells (ln HCMV-DNA, ln C7-HRP, and ln C10/11). Spearman’s correlations were performed to determine the association between ln HCMV-DNA and ln C7-HRP, and between ln HCMV-DNA and ln C10/11. **Significant at *p* < 0.001 by Spearman’s rank correlation coefficient. ^†^Correlation coefficient. *HCMV* human cytomegalovirus, *HRP* horseradish peroxidase

We examined the relationship between the HCMV-DNA copy number and other HCMV tests. No association between nuclear inclusion bodies on H&E staining and HCMV mucosal PCR was observed (Fig. 2A), while we found a significant association between the HCMV-DNA copy number and the positivity of IHC (*p* < 0.01) (Fig. 2B). Regarding HCMV antigenemia, there was a positive correlation between HCMV-DNA copy number and the number of positive cells for C7-HRP (*p* < 0.001, *r*_*s*_ = 0.58) (Fig. 3A) and C10/11 (*p* < 0.001, *r*_*s*_ = 0.56) (Fig. 3B).

**Fig. 4:**
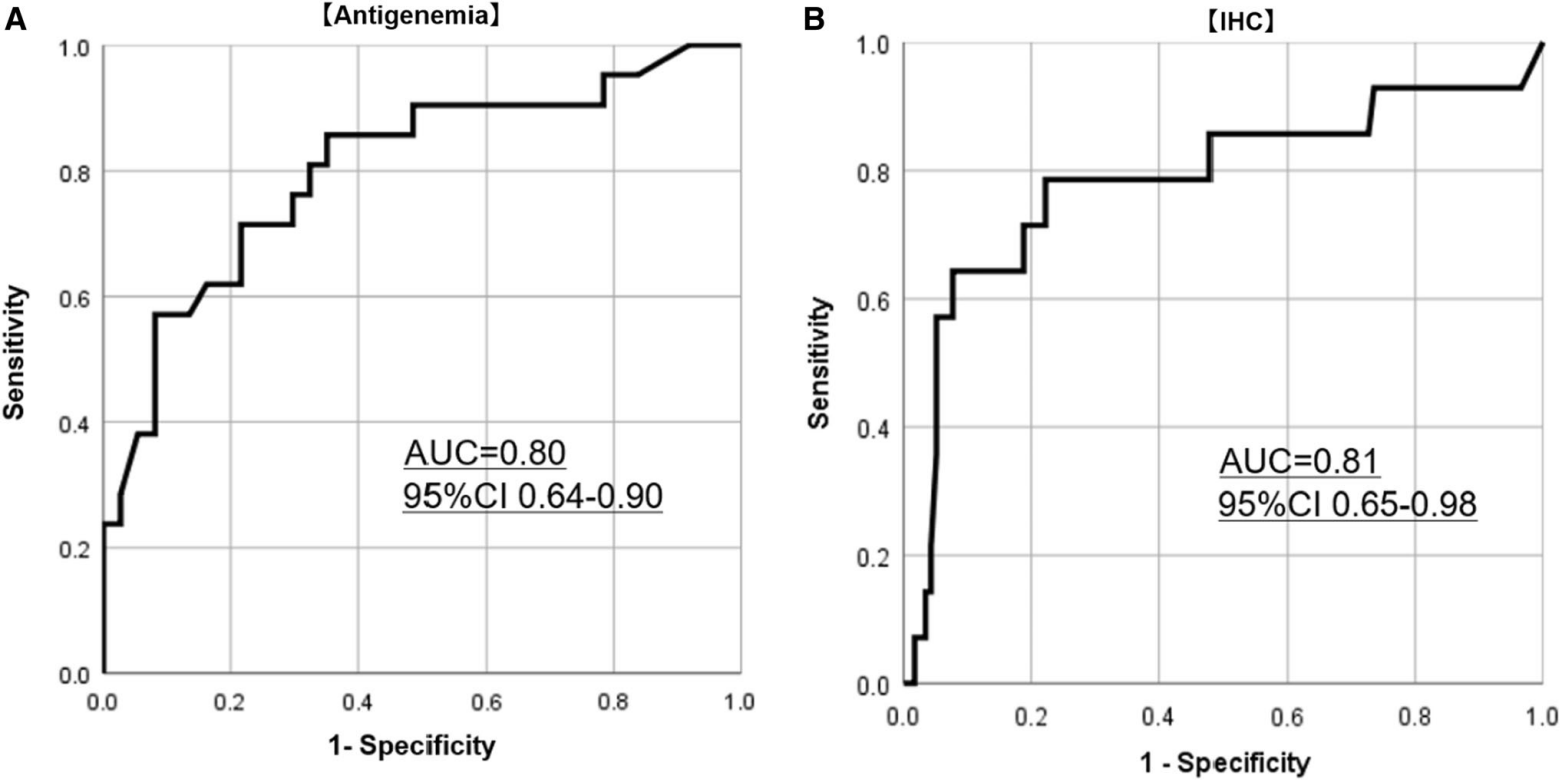
ROC curve analysis. ROC curves for **A** antigenemia (AUC = 0.80, Sensitivity = 71.4%, Specificity = 78.4%) and **B** IHC (AUC = 0.81, Sensitivity = 78.6%, Specificity = 77.8%). *ROC* Receiver operating characteristic, *IHC* Immunohistochemistry, *AUC* Area under the curve, 95% CI 95% confidence interval

Next, we generated a receiver operating characteristic (ROC) curve and calculated the area under the curve to determine the best discriminating level of HCMV-DNA copy number for predicting an HCMV antigenemia-positive cell. ROC curve analysis confirmed 1,300 copies/μg as the best diagnostic cut-off value of HCMV-DNA copy number for a positive result of antigenemia (area under the curve = 0.80, 95% CI 0.64–0.90, sensitivity 71.4% and specificity 78.4%) (Fig. 4A). For IHC, ROC curve analysis confirmed 1,650 copies/μg as the best diagnostic cut-off value of HCMV-DNA copy number for a positive result of IHC (area under the curve = 0.81, 95% CI 0.65–0.98, sensitivity 78.6% and specificity 85.7%) (Fig. 4B).

We compared HCMV-DNA copy numbers between the groups with positive and/or negative results on the antigenemia and histopathological tests (Table S2). Twenty-eight patients with refractory UC who were negative for both the antigenemia and histopathological tests had lower levels of mucosal HCMV-DNA than those who were positive for either one or both (Fig. S2). Among the 28 patients, 13 received antiviral agents, and 15 did not. The clinical outcome of these patients showed that one patient underwent total colectomy in each group (Table S3).

### Relationship between the HCMV-DNA copy number and endoscopic findings

**Fig. 5:**
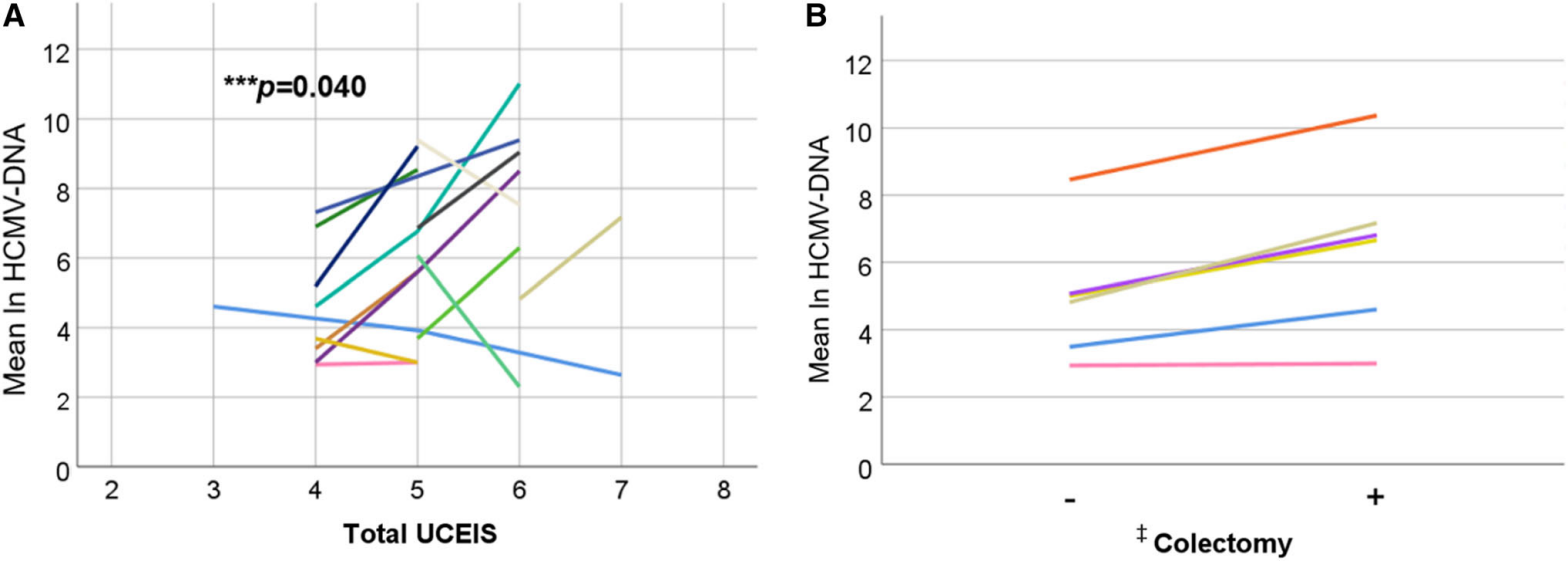
The relationship between HCMV-DNA copy numbers and **A** total UCEIS or **B** colectomy using a mixed model^†^. The vertical axis is the natural log-transformed copy number of HCMV-DNA (ln HCMV-DNA). Each colored line represents changes in individual patients. When the UCEIS scores were the same for each individual patient, the values of ln HCMV-DNA were averaged. ^†^Mixed model is a statistical model containing both fixed and random effects, where measurements are made on clusters of related statistical units. ^‡^Only patients who underwent colectomy were included in the analysis. *HCMV* human cytomegalovirus, *UCEIS* Ulcerative Colitis Endoscopic Index of Severity

We examined the relationship between the HCMV-DNA copy number and endoscopic findings (MES and UCEIS). Patients with MES 3 tended to have a higher HCMV-DNA copy number than those with MES 1 and 2 (Fig. S3A). For the UCEIS, the total UCEIS score was correlated with the HCMV-DNA copy number (*p* = 0.014, *r*_*s*_ = 0.22) (Fig. S3B). Among the items included in the UCEIS, the bleeding score was also correlated with the HCMV-DNA copy number (*p* = 0.013, *r*_*s*_ = 0.22) (Fig. S3C). However, there was no significant correlation between the HCMV-DNA copy number and the other two items, erosion/ulcer and vascular permeability (Fig. S3D, E). Moreover, an analysis using a mixed model that takes into account correlations for each individual patient showed a positive correlation between the total UCEIS score and HCMV-DNA copy number (*p* = 0.040) (Fig. 5A). Furthermore, more cases led to colectomy as the HCMV-DNA copy number increased (Fig. 5B). On the other hand, there was no similar correlation for MES (no figure).

## Discussion

This is the first study to evaluate the relationship between HCMV-DNA copy numbers in colonic tissues and other HCMV tests or endoscopic scores. We found a significant correlation between HCMV-DNA copy number in colonic tissues and both the antigenemia assay and histopathological tests by IHC in UC patients with concomitant HCMV colitis. Regarding endoscopic scores, HCMV-DNA copy number by mucosal PCR correlated with both the total UCEIS score and the bleeding score. Moreover, a positive correlation between the change in the total UCEIS score and HCMV-DNA copy number for UC patients taking multiple mucosal PCR tests.

Generally, HCMV is contracted during childhood and can persist as a lifelong latent infection, reactivating under inflammatory conditions in an immunosuppressed host [1, 2]. HCMV infection is involved in active UC patients who are refractory to steroids and immunosuppressive drugs [3, 5, 18]. Previous reports showed that steroids are predisposing factors for HCMV reactivation by suppressing anti-HCMV T-cell specific function and immunoglobulin production via B-cells, and by directly activating viral replication [19–21]. In this study, patients treated with PSL had a higher HCMV-DNA copy number than those treated without PSL. In patients with acute steroid-resistant UC, HCMV infection should be excluded before increasing immunosuppressive drugs [22]. However, the clinical features of HCMV enterocolitis are sometimes indistinguishable from UC relapse [23–25].

Histological examination, including H&E staining and IHC, on biopsy from colonic tissues is the gold standard for diagnosing HCMV enterocolitis, which yields high specificity but variable sensitivity [1, 6, 7]. IHC has 22.1–30.0% higher sensitivity than H&E staining for diagnosing HCMV infection in IBD patients [26–28]. In the present study, the positive rate of HCMV antigen by IHC correlated with the HCMV-DNA copy number.

Another standard method for detecting HCMV infection is the HCMV antigenemia assay. Previous reports demonstrated that the antigenemia assay has a high specificity for detecting HCMV and a significant association with the subsequent colectomy rate in UC patients with concomitant HCMV enterocolitis [8–11]. However, the sensitivity for diagnosing HCMV gastrointestinal diseases is only 47.0–67.3%, suggesting that the antigenemia assay alone is considered insufficient for early detection of HCMV [8–11].

Yoshino et al. reported that a mucosal quantitative real-time PCR assay for detecting HCMV-DNA in the gastrointestinal tract was useful for the early and accurate diagnosis of HCMV infection in active UC patients refractory to immunosuppressive therapies [12]. Roblin et al. reported that UC patients with an HCMV-DNA load higher than 250 copies/mg in colonic tissue required early antiviral treatment [29]. However, mucosal PCR assays cannot be available in all medical institutions, and most physicians should decide to start antiviral treatment based on the results of antigenemia assays or histopathological tests in clinical practice. In the present study, we found a positive correlation between HCMV-DNA copy number and both HCMV antigenemia assay and IHC positivity. ROC analysis showed that 1300 (AUC = 0.80, 95% CI = 0.64–0.90) and 1,650 (AUC = 0.81, 95% CI = 0.65–0.98) copies/µg of HCMV-DNA were the best diagnostic values to detect HCMV antigenemia-positive cells and IHC positivity, respectively. These data suggest that positivity for HCMV antigenemia and IHC signify the increase of HCMV-DNA in the inflamed colon to decide the intervention of antiviral treatment for HCMV.

Conversely, among UC patients who were negative for both the antigenemia and histopathological tests, the HCMV-DNA copy numbers were significantly lower than those in the other groups. In this group, the median HCMV-DNA copy number was 300 copies/μg (interquartile range: 39–810 copies/μg); however, there were no significant differences in HCMV-DNA copy numbers and the clinical outcome regardless of the antiviral treatment. These data suggest that a group of patients with a low mucosal HCMV-DNA copy number (less than 800 copies/μg) during treatment is more likely to be controlled with immune regulating therapy alone. However, given that the inflammatory control was poor during treatment, even in patients with a low mucosal HCM-DNA copy number at diagnosis, we must consider that exacerbation of HCMV infection can modify the disease state.

Regarding endoscopic findings, Suzuki et al. reported that irregular punched-out or longitudinal ulcers were specific for HCMV infection in UC patients [24]. On the other hand, the endoscopic findings of HCMV colitis are variable and sometimes do not reveal specific features [12, 30]. The present study also showed that the endoscopic scores varied even with the same amount of HCMV-DNA copy numbers. Taken together, previous and current data suggest that the heterogeneity of the immune response to HCMV and mucosal cytokine pattern in each UC patient could contribute to endoscopic features of UC with concomitant HCMV infection [5, 31–33]. To date, there have been no reports on the relationship between HCMV enterocolitis and endoscopic scores for MES or UCEIS in IBD patients. In this study, the total UCEIS score and the bleeding score were correlated with the HCMV-DNA copy numbers. Moreover, when we focused on patients who underwent multiple HCMV mucosal PCR tests, we found a significant correlation between changes in HCMV-DNA and those in the total UCEIS. At the beginning of HCMV reactivation, HCMV can infect the vascular endothelium and cause ischemic damage to the mucosa, which results in gastrointestinal bleeding [34]. Previous reports have shown that gastrointestinal bleeding is the most frequent symptom of HCMV enterocolitis [34, 35]. Thus, in UC patients refractory to immune suppressive therapy who had a higher total UCEIS score, severe bleeding score, and increasing total UCEIS score during the clinical course, we should consider the involvement of HCMV reactivation in the colon. Furthermore, HCMV-DNA copy number tended to increase in cases who led to colectomy. These results suggest that early initiation of antiviral therapy may avoid the risk of colectomy in cases with elevated UCEIS scores.

There are several limitations to this study. First, because of the retrospective study design, not all patients underwent antigenemia assays and histopathological tests with IHC. Additionally, not all HCMV antigenemia assays were performed on the same date as the biopsy for mucosal PCR. Since the difference in dates between HCMV antigenemia and mucosal PCR tests was at most 7 days, of which 71% were within 3 days, the initiation of therapeutic agents (i.e., steroids, biologics, and antiviral agents) might have affected the value of antigenemia in cases where the antigenemia was measured after mucosal PCR was performed. Second, whether to measure HCMV-DNA was made by each attending physician, which could be at risk for selection bias.

In conclusion, our data showed that the antigenemia assay and histopathological test with IHC could help estimate the HCMV-DNA copy number in colonic tissues. Furthermore, the changes in UCEIS score in each individual may be helpful for decision-making in UC patients with concomitant HCMV infection. Additional study is required to establish an accurate diagnostic scoring system for the early diagnosis of HCMV infection in UC patients with a combination of HCMV tests and endoscopic score.

## Supplementary Information

Below is the link to the electronic supplementary material.

Supplementary file1 (DOCX 925 KB)

Supplementary file2 (DOCX 540 KB)

## Data Availability

The data that support the findings of this study are available from the corresponding author on reasonable request due to privacy or ethical restrictions.

## Abbreviations

HCMV: Human cytomegalovirus
H&E: Hematoxylin and eosin
IHC: Immunohistochemistry
IM: Immunomodulator
MES: Mayo endoscopic subscore
PCR: Polymerase chain reaction
PSL: Prednisolone
Tac: Tacrolimus
UC: Ulcerative colitis
UCEIS: Ulcerative Colitis Endoscopic Index of Severity
