# Correction: The association between antigenemia, histology with immunohistochemistry, and mucosal PCR in the diagnosis of ulcerative colitis with concomitant human cytomegalovirus infection

**DOI:** 10.1007/s00535-023-01979-8

**Published:** 2023-03-08

**Authors:** Tsukasa Yamakawa, Hiroki Kitamoto, Masanori Nojima, Tomoe Kazama, Kohei Wagatsuma, Keisuke Ishigami, Shuji Yamamoto, Yusuke Honzawa, Minoru Matsuura, Hiroshi Seno, Hiroshi Nakase

**Affiliations:** 1 Department of Gastroenterology and Hepatology, Sapporo Medical University School of Medicine, S-1, W-16, Chuo-Ku, Sapporo, 060-8543 Japan; 2 Department of Gastroenterology and Hepatology, Kyoto University School of Medicine, Kyoto, Japan; 3 Center for Translational Research, Institute of Medical Science Hospital, University of Tokyo, Tokyo, Japan; 4 Department of Gastroenterology and Hepatology, Kyorin University School of Medicine, Tokyo, Japan

**Correction: Journal of Gastroenterology (2022) 58:44–52** 10.1007/s00535-022-01931-2

In the original publication of the article, the first author’s name should be “Tsukasa Yamakawa” and not “Tsukasa Yamawaka”.

