# Supplementary figures and images for: The association between antigenemia, histology with immunohistochemistry, and mucosal PCR in the diagnosis of ulcerative colitis with concomitant human cytomegalovirus infection
Source: J Gastroenterol. 2022 Oct 26;58(1):44–52. doi: 10.1007/s00535-022-01931-2 (PMC9825535; doi:10.1007/s00535-022-01931-2)

(Supplementary Table 1)


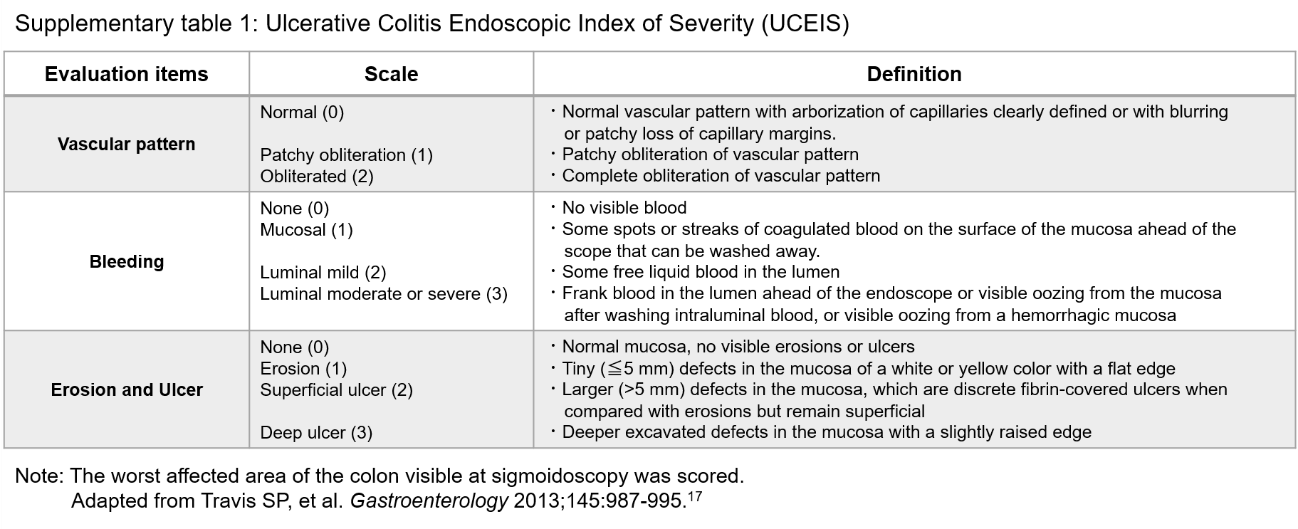


(Supplementary Table 2)


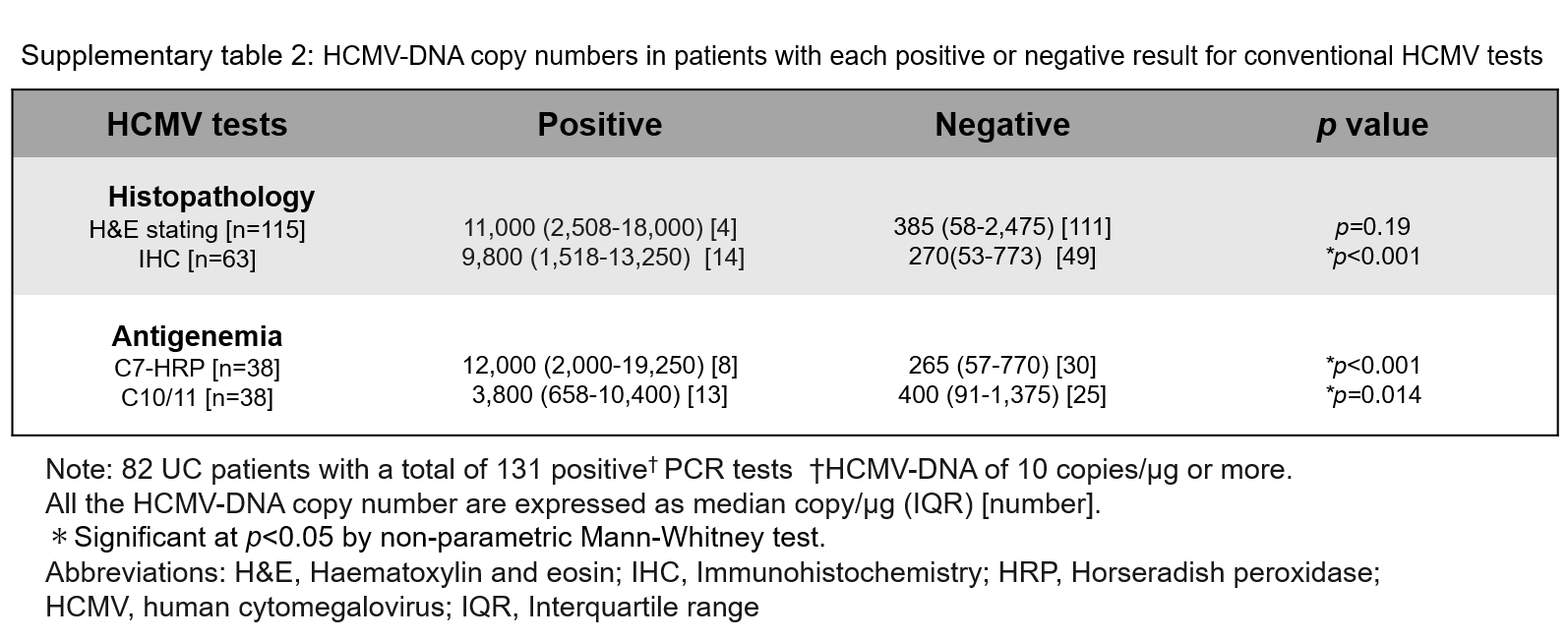


（Supplementary Table 3）


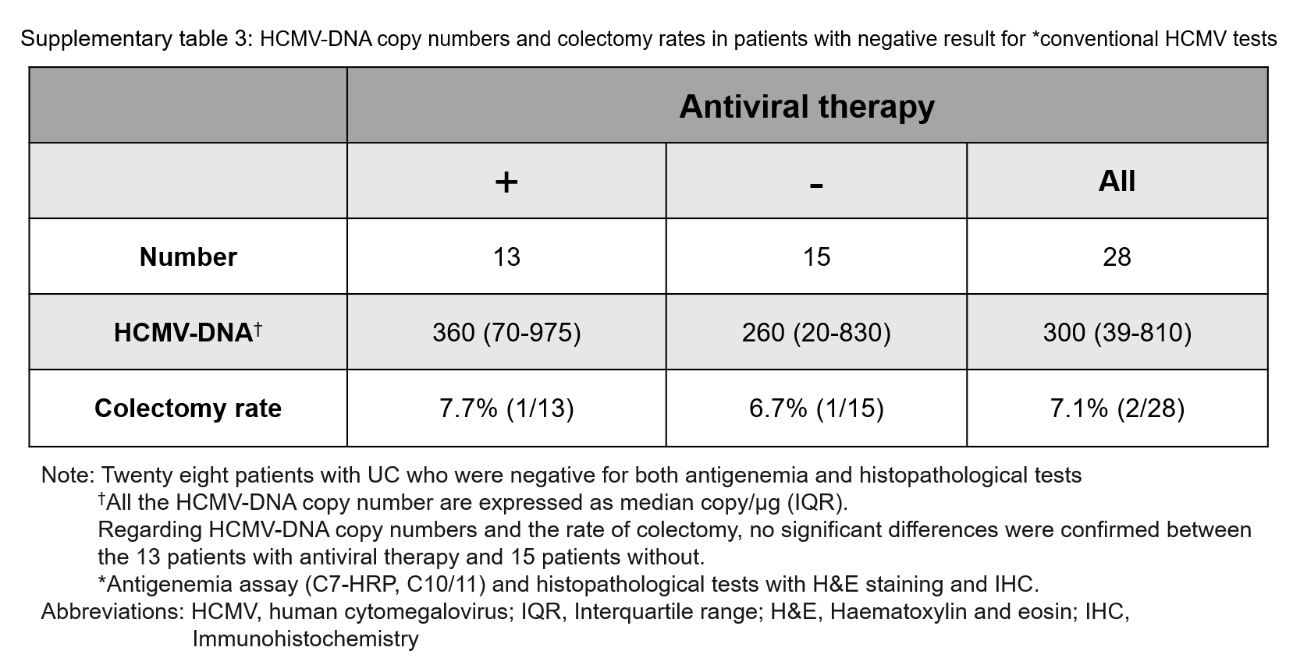

Supplement: Supplementary file 1 — Supplementary file1 (DOCX 925 KB) [file 535_2022_1931_MOESM1_ESM.docx]
