# Supplementary material for: The association between antigenemia, histology with immunohistochemistry, and mucosal PCR in the diagnosis of ulcerative colitis with concomitant human cytomegalovirus infection
Source: J Gastroenterol. 2022 Oct 26;58(1):44–52. doi: 10.1007/s00535-022-01931-2 (PMC9825535; doi:10.1007/s00535-022-01931-2)
Supplement: Supplementary file 2 — Supplementary file2 (DOCX 540 KB) [file 535_2022_1931_MOESM2_ESM.docx]

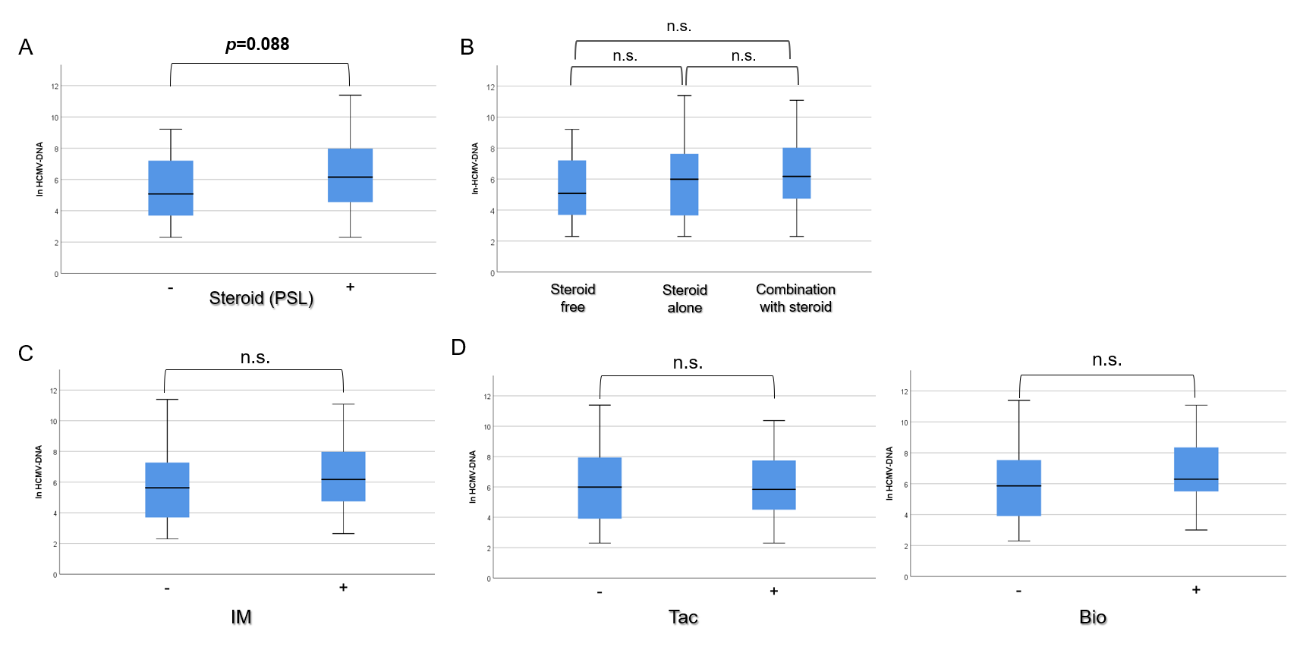


(Supplementary Figure 1)

**Supplement figure 1:** The relationship between the medications and HCMV-DNA copy number. (A, B) PSL, (C) IM, (D) Tac, (E) Bio

Note: The vertical axis is the natural log-transformed copy number of HCMV-DNA. (ln HCMV-DNA)

＊Significant at *p*<0.05 by non-parametric Mann–Whitney U test.

Abbreviation: HCMV, human cytomegalovirus; PSL, prednisolone; IM, immunomodulator; Tac, tacrolimus; Bio, biologics; n.s., not statistically significant


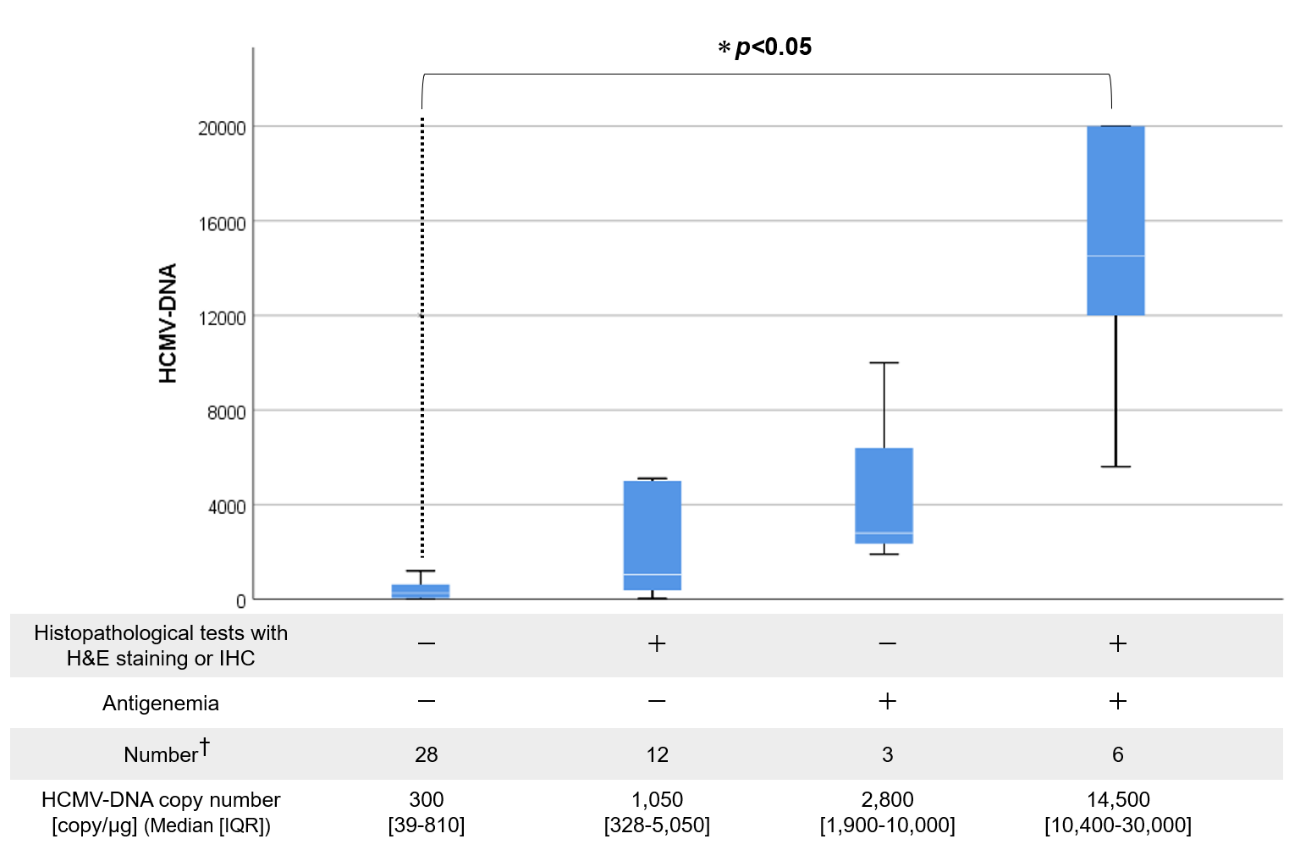


(Supplementary Figure 2)

**Supplement Figure 2**: HCMV-DNA copy numbers in the UC patients when antigenemia and histopathological tests with H&E staining or IHC were positive or negative, both positive and negative.

Note: †Forty-eight cases in which both antigenemia and histopathological tests with H&E staining or IHC were examined. All the HCMV-DNA copy numbers are expressed as median [IQR].

＊Significant at p<0.05 by non-parametric Mann–Whitney U test.

Abbreviations: H&E, Hematoxylin and Eosin staining; IHC, Immunohistochemistry


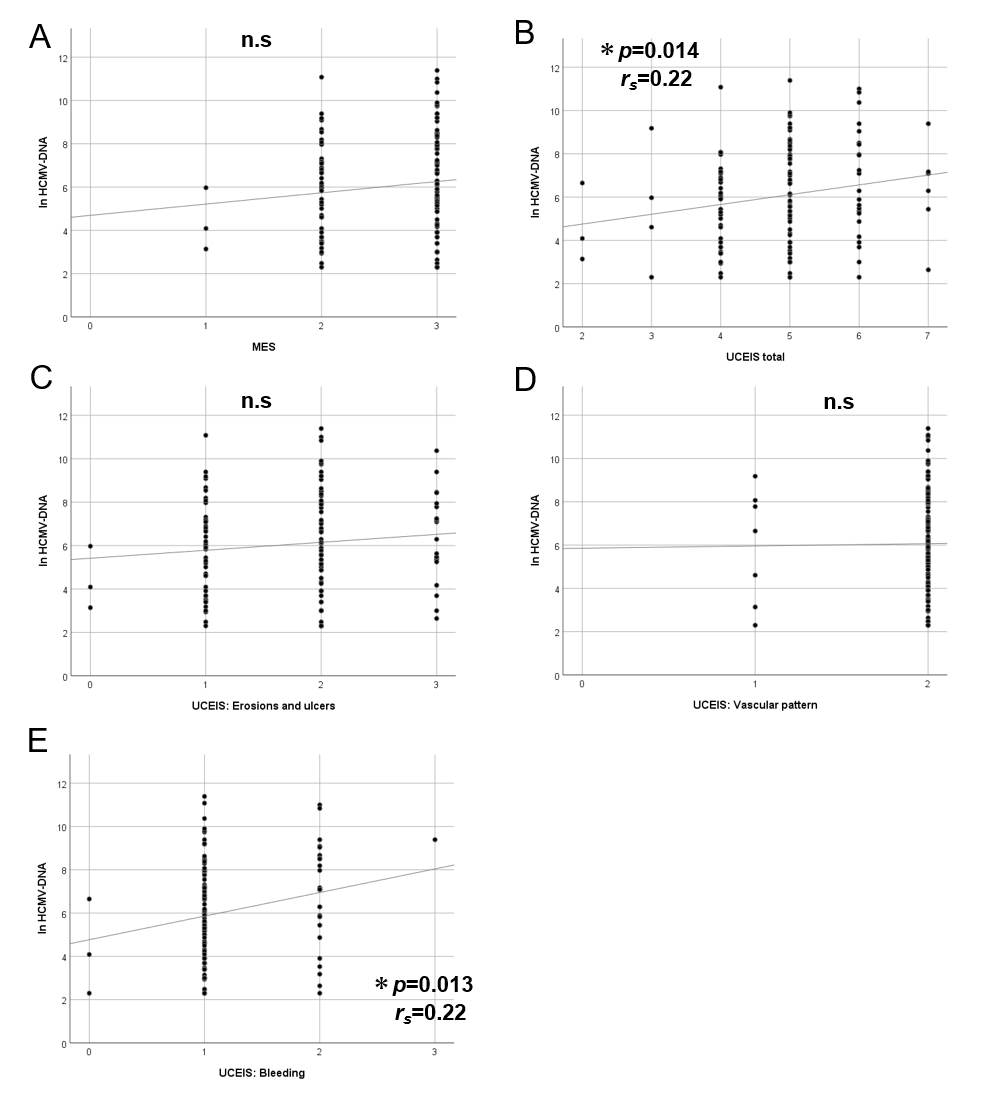


(Supplementary Figure 3)

**Supplement figure 3:** The relationship between HCMV-DNA copy number and endoscopic findings. (A) MES score, (B) total UCEIS score, (C) UCEIS: Erosion and ulcer, (D) UCEIS: Vascular pattern, (E) UCEIS: Bleeding

Note: The vertical axis is the natural log-transformed copy number of HCMV-DNA (ln HCMV-DNA) Spearman’s correlations were performed to examine the relationship between HCMV-DNA copy numbers and endoscopic findings.

Abbreviations: HCMV, human cytomegalovirus; MES, Mayo endoscopic subscore; UCEIS, Ulcerative Colitis Endoscopic Index of Severity; n.s., not statistically significant
